# Mirtazapine versus Megestrol in the Treatment of Anorexia–Cachexia Syndrome in Patients with Advanced Cancer: A Randomized, Double-Blind, Controlled Phase II Clinical Trial

**DOI:** 10.3390/cancers15143588

**Published:** 2023-07-12

**Authors:** Olga Laura Sena Almeida, Eduardo Ferriolli, Roberta Cristina Cintra Taveira, Meire Gallo Rosenburg, Daniela Dalpubel Campanari, Natália Maira da Cruz Alves, Karina Pfrimer, Liane Rapatoni, Fernanda Maris Peria, Nereida K. C. Lima

**Affiliations:** 1 Department of Internal Medicine, Ribeirão Preto Medical School, University of São Paulo, Ribeirão Preto 14049-900, SP, Brazil; 2 Department of Medical Imaging, Hematology and Clinical Oncology, Ribeirão Preto Medical School, University of São Paulo, Ribeirão Preto 14049-900, SP, Brazil

**Keywords:** mirtazapine, megestrol, anorexia, cachexia, cancer

## Abstract

**Simple Summary:**

This study compared mirtazapine with megestrol in the management of cancer-related anorexia–cachexia syndrome in patients with advanced cancer. This is a randomized, double-blind, controlled clinical trial involving patients with advanced cancer and anorexia–cachexia syndrome. Participants received mirtazapine 30 mg/day or megestrol 320 mg/day for eight weeks. Fifty-two patients were randomized. There was weight gain in 52% of the participants in the megestrol group and in 38% in the mirtazapine group after four weeks (*p* = 0.040). Appetite improved in 92% of the participants in the megestrol group and in 56% in the mirtazapine group after eight weeks (*p* = 0.007). In the sub-analysis by sex, women showed improvement in appetite (*p* < 0.001) and weight gain (*p* < 0.005) in the mirtazapine group, which was not observed in men. Mirtazapine appears to be inferior to megestrol in weight and appetite improvement. However, there may be a difference in the therapeutic response between sexes.

**Abstract:**

This study compared mirtazapine with megestrol in the management of cancer-related anorexia–cachexia syndrome in patients with advanced cancer. A randomized, double-blind, controlled clinical trial involving patients with advanced cancer and anorexia–cachexia syndrome was performed. Participants received mirtazapine 30 mg/day or megestrol 320 mg/day for eight weeks. The primary endpoint was the effect of mirtazapine on weight gain and the secondary endpoints were its effect on appetite, muscle strength, physical performance, body composition, adverse events, and medication adherence. Linear regression model with mixed effects was applied and a significance level of 5% was adopted. Fifty-two patients were randomized. Mean age was 65.8 ± 8.4 years. There was weight gain in 52% of the participants in the megestrol group and in 38% in the mirtazapine group after four weeks (*p* = 0.040). Appetite improved in 92% of the participants in the megestrol group and in 56% in the mirtazapine group after eight weeks (*p* = 0.007). In the sub-analysis by sex, women showed improvement in appetite (*p* < 0.001) and weight gain (*p* < 0.005) in the mirtazapine group, which was not observed in men. Mirtazapine appears to be inferior to megestrol in weight and appetite improvement. However, there may be a difference in the therapeutic response between sexes.

## 1. Introduction

Cancer-associated cachexia is a multifactorial condition characterized by continuous loss of muscle mass, with or without loss of fat mass, which cannot be completely reversed by conventional nutritional support, and which leads to progressive functional impairment [1]. Anorexia and weight loss are the two major symptoms of cancer-associated cachexia [2]. However, the relationship between anorexia and weight loss remains unclear and evidence suggests that both occur independently [3].

The anorexia–cachexia syndrome (ACS) reduces tolerance to cancer treatment, with reductions in the dose of chemotherapy drugs and delays or suspension of treatment, as well as increases susceptibility to infections and other complications [4,5]. The incidence and severity of ACS increase with the progression of metastatic cancer disease [6].

Despite its clinical importance, cancer-related ACS remains an underestimated and untreated condition [7]. Several randomized clinical trials involving pharmacological agents have been developed [8]. Anamorelin and the anti-GDF15 monoclonal antibody appear to be promising drugs [8,9], but there is no single FDA-approved gold standard agent for the management of ACS in patients with cancer [8]. In 2020, the American Society of Clinical Oncology (ASCO) published a guideline on the therapeutic management of cancer-related ACS [10]. As for pharmacological management, evidence remains insufficient to strongly endorse any pharmacological agent. However, physicians may offer a short-term trial of a progesterone analogue or a corticosteroid for patients with loss of appetite and/or weight loss. The choice of agent and duration of treatment depend on the goals of treatment and the assessment of risk versus benefit [10].

Considering the scarcity of pharmacological measures for the treatment of cancer-related ACS and the impact of this condition on the survival and quality of life of cancer patients, it is necessary to invest in studies that can contribute to the rational and effective treatment of this condition, reducing its impact on morbidity and mortality of cancer patients with advanced disease. Mirtazapine may be a promising therapeutic option. It is a widely used tetracyclic antidepressant with serotonergic and noradrenergic action, with a low frequency of adverse effects, which induces weight gain and increases food intake [11,12,13,14,15]. The increase in appetite with consequent weight gain may be due to the blockade of postsynaptic 5-HT2 and 5-HT1B receptors, changing the secretion of neuropeptide Y, which is involved in appetite stimulation [16,17]. In addition, mirtazapine also acts in blocking 5-HT3 [12] and H1 [18] receptors, promoting changes in serum cytokine levels [19].

A pilot study with two doses of mirtazapine (15 mg or 30 mg daily), involving patients with advanced cancer, pain, and other limiting symptoms, suggested significant improvement in quality of life, particularly in terms of weight gain and food intake [20]. An open-label, uncontrolled clinical trial involving non-depressed patients with cancer with metastatic disease also suggested that mirtazapine may be a promising therapeutic option for the control of cancer-associated ACS, with weight gain of at least one kilogram in 24% of patients included after four weeks of treatment [21].

This study aimed to evaluate the effect of mirtazapine compared to megestrol acetate in the management of cancer-related ACS in patients with advanced disease.

## 2. Materials and Methods

### 2.1. Eligibility Criteria

Inclusion criteria were age ≥ 50 years; confirmed diagnosis of malignant neoplasm by histopathology; progression of disease both locally and metastatic; complaint of anorexia graded by the patient as ≥2 by the Edmonton Symptom Assessment Scale (ESAS); weight loss > 5% in the last six months or >2% in the last two months associated with a body mass index (BMI) < 20 kg/m^2^ or reduced muscle mass [1]; life expectancy ≥ 30 days by the Palliative Prognostic Score; performance status ≥ 60% according to the Karnofsky Performance Status scale.

Exclusion criteria were diagnosis of depression or use of antidepressant therapy within the last four weeks with a score ≥ 12 in the items related to depression on the Hospital Anxiety and Depression scale; use of unstable doses of corticosteroids; moderate renal and/or hepatic dysfunction; decompensated hypothyroidism; uncorrected electrolyte disturbances; central nervous system (CNS) metastases; mechanical obstruction of the gastrointestinal tract; clinically voluminous ascites and generalized edema; persistent and uncontrolled nausea and/or vomiting; inability to ingest medications orally; use of nasoenteral tube; polycythemia; previous thromboembolic event; acute myocardial infarction or cerebrovascular event within less than six months; decompensated heart failure; use of pacemaker; poorly controlled type 2 diabetes mellitus or systemic arterial hypertension; HIV infection; moderate to severe cognitive deficit; institutionalization; hospital admission at the time of the initial assessment; current use of megestrol or mirtazapine; allergy to the studied medications; refusal to participate.

### 2.2. Study Design and Participants

This is a phase II, randomized, double-blind, controlled clinical trial, which was developed in the outpatient clinical oncology service of a tertiary-level university hospital.

To calculate the sample size, weight gain was the outcome used (1.06 ± 1.95 kg) [22], with 80% power and significance level of 5%. Forty patients were required to complete the study and, considering an estimated sample loss of 20% due to the severity of the underlying disease, 52 patients were randomized.

Electronic randomization was performed on the “sealed envelope” website (https://www.sealedenvelope.com/simple-randomiser/v1/lists, accessed on 5 March 2019) in six blocks with eight patients and one block with four, in which half of the patients received mirtazapine and the other half megestrol.

The study was double-blind and the researchers, participants, and those responsible for data analysis did not know about the distribution of patients in the groups. Medications were stored in identical opaque white bottles that did not allow identification. There was only one person responsible for the randomization and preparation of the vials.

This study was approved by the local Research Ethics Committee and registered on the ClinicalTrials.gov platform (NCT03283488). A free and informed consent form was signed by all the study participants.

### 2.3. Intervention and Assessments

Participants were randomized to receive mirtazapine or megestrol (15 mg and 160 mg tablets, respectively) and instructed to initially take one tablet at night. From the second week onwards, if there was good tolerance, they were instructed to ingest two tablets at night until completing eight weeks of follow-up. Those who interrupted the use of medication for more than two consecutive days would be excluded [21]. At the end of the follow-up period, participants who wished to continue using the medication had the option of doing so. For those who did not, the drug was reduced and discontinued one week after the end of the intervention. The doses of mirtazapine and megestrol used were based on previous studies [21,23]. Patients who were already using oral nutritional supplementation before the intervention were instructed to continue using it, without changing the dose during the follow-up period.

There was an in-person assessment at the fourth and eighth weeks of follow-up, with telephone contact in the remaining weeks for the assessment of adverse events and medication adherence.

All included participants were evaluated for the following variables: general characteristics; performance status by the Karnofsky Performance Status scale; anthropometric assessment; appetite by the Edmonton Symptom Assessment Scale; muscle strength by the handgrip test performed with a manual hydraulic dynamometer (Saehan, model SH 5.001, Changwon-si, South Korea); physical performance by gait speed test; body composition by dual energy X-ray absorptiometry (DXA, LUNAR device, Prodigy Model). All assessments were performed before and after eight weeks of the beginning of the intervention, except weight and appetite, which were also assessed after four weeks.

The anthropometric assessment was carried out using weight and height measurements, with the calculation of the BMI by the ratio of weight to height squared. Data were collected according to the International Standards for Anthropometric Assessment protocol [24].

To assess appetite and its intensity, the Edmonton Symptom Assessment System scale was used. This tool allows the objective classification of symptom intensity, on a scale ranging from zero (absence of symptoms) to ten (worst intensity) [25].

The handgrip test was performed in accordance with the guidelines of the American Society of Hand Therapists [26]. Strength was measured three times in the dominant hand, with one minute of rest between attempts, during a verbally stimulated effort for six seconds, the highest value of the three measures being considered. The cutoff point used was 27 kgF for men and 16 kgF for women. Participants with measurements below these cutoffs were classified as having reduced muscle strength [27].

The gait speed test was performed to assess physical performance as it is associated with functional disability and general health decline [27]. The test was performed by asking the participant to cover a distance of four meters, at their usual speed and with a gait device if they used it. The average of two measurements was considered [28]. Participants with gait speed ≤ 0.8 m/s were classified as having reduced gait speed and low physical performance [27].

For the evaluation of body composition by DXA, the total body protocol was used in the specific software of the equipment. The variables used were total mass, fat mass, fat-free mass, appendicular lean mass, and appendicular lean mass index. The cutoff used for the appendicular lean mass index (ALMI) was 7.0 kg/m^2^ for men and 6.0 kg/m^2^ for women [27]. Participants who had ALMI levels below the cutoffs used were classified as having reduced muscle mass [27]. Participants who had reduced muscle strength by handgrip and reduced muscle mass by DXA were classified as sarcopenic [27].

### 2.4. Medication Adherence

Medication adherence was assessed by a weekly interview with direct questioning about ingestion, by checking a diary of medication use and by manually counting the tablets when the bottles were returned. The number of remaining tablets was compared with the prescribed therapeutic regimen. Patients who used 80 to 100% of the estimated number of tablets for the evaluated time interval were classified as adherent [29].

### 2.5. Outcomes

The primary outcome was weight change, which was classified into three categories: weight improvement as a gain ≥1 kg, weight maintenance as a loss of <500 g or a gain of <1 kg, and weight loss as a loss of ≥500 g [21].

Secondary outcomes were changes in appetite, muscle strength, physical performance, and body composition. Changes in appetite were also classified into three categories: improvement in appetite as a decrease of ≥2 points in the ESAS score, maintenance of appetite as an improvement or worsening of 1 point, and worsening of appetite as a deterioration of ≥2 points [21].

### 2.6. Statistical Analysis

For comparisons between groups and times regarding quantitative variables, linear regression with mixed effects (random and fixed effects), which consider the presence of repeated measures among individuals, was applied. The assumptions of normality and homoscedasticity of the residuals were verified and validated through normality tests and graphs such as histogram, quantile–quantile, and dispersion between residuals and predicted. Comparisons made regarding levels of lack of appetite were analyzed using an ordinal multinomial logistic regression model with repeated measures, given the ordinal characteristic of the outcome variable. The comparison between groups and times regarding binary variables was performed by Poisson regression with robust variance and logarithmic linkage function, which allowed estimating the corresponding relative risks. Comparison between groups regarding medication intake was analyzed by quantile regression. For all comparisons, raw models were analyzed and adjusted for possible confounding variables (sex, age, disease stage, weight loss, disease duration, previous surgical treatment, previous radiotherapy). A significance level of 5% was adopted. Analyses were performed using SAS 9.4 software.

## 3. Results

### 3.1. Patients and Randomization

**Figure 1:**
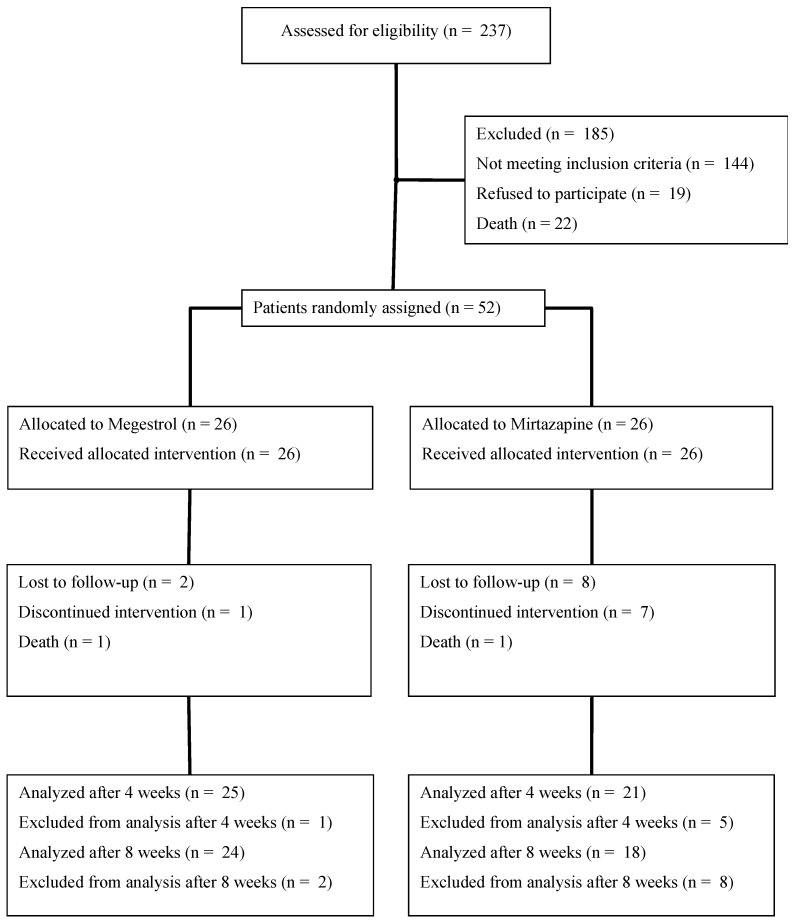
CONSORT Diagram.

Data collection took place from March 2019 to February 2022. During this period, 237 patients were eligible and 52 were randomized with 26 in each group (Figure 1).

The mean age of the participants was 67.0 ± 8.6 years in the megestrol group and 64.5 ± 8.2 years in the mirtazapine group (*p* = 0.289). The mean weight loss in six months was 12 ± 6% in the megestrol group and 11 ± 4% in the mirtazapine group (*p* = 0.737). There was no difference between the groups regarding general characteristics (Table 1), nor regarding the mean weight and appetite score before the intervention (*p* = 0.510 and 0.727, respectively).

### 3.2. Outcomes

#### 3.2.1. Weight and Appetite

Weight improved in 52% of the participants in the megestrol group and in 38% of the participants in the mirtazapine group after four weeks (*p* = 0.040), but this difference was not maintained after eight weeks, with weight maintenance or improvement in 50% of the participants in both groups (*p* = 0.166). Appetite improved after eight weeks in 92% of the participants in the megestrol group and in 56% of the participants in the mirtazapine group (*p* = 0.007). Participants in the megestrol group had, on average, a 169% greater chance of gaining weight after four weeks (*p* = 0.032) and an 85% greater chance of improving their appetite after eight weeks (*p* = 0.008), compared to the mirtazapine group (Table 2).

There was no intra and intergroup difference in weight and BMI means in any of the study moments. However, when the analysis was performed by sex, women had significant weight gain after the intervention in the mirtazapine group, with an estimated difference of 1.92 kg (95% CI −3.54 to −0.31, *p* = 0.020) and 2.48 kg (95% CI −4.15 to −0.81, *p* = 0.004) after four and eight weeks, respectively. On the other hand, this difference was not observed in men. Table 3 describes the median weight and BMI, by sex, of participants in both groups, before and after the intervention.

The participants in both groups were more likely to have a worse appetite before the intervention when compared to four and eight weeks (*p* < 0.001), with a greater chance of appetite improvement in the megestrol group after the intervention (*p* = 0.022). In the sub-analysis by sex, there was a greater chance of appetite improvement in men in the megestrol group after the intervention, which was not observed in men in the mirtazapine group. On the other hand, women in both groups were more likely to have improved their appetite after the intervention, with no difference between groups (Table 4).

#### 3.2.2. Body Composition, Muscle Strength, and Physical Performance

In the assessment of body composition, women in the mirtazapine group had higher appendicular lean mass (ALM) before and after the intervention, compared to women in the megestrol group (*p* = 0.035 and 0.034, respectively), with a 74% greater chance of not having confirmed sarcopenia after the intervention (RR 0.74, 95% CI 0.57 to 0.96, *p* = 0.025). There was an increase in fat mass after the intervention in this group, without a difference in intra or intergroups. As for muscle strength, all women in both groups had normal handgrip strength before and after the intervention, with no intra and intergroup differences. There was a significant reduction in handgrip strength in men in the megestrol group (1.87 kgF, 95% CI 0.12 to 3.62, *p* = 0.037), as well as in the mirtazapine group (4.22 kgF, 95% CI 1.69 to 6.74, *p* = 0.002), with more pronounced worsening in men in the mirtazapine group (5.05 kgF, 95% CI 0.42 to 9.68, *p* = 0.033). Regarding physical performance, there was a significant worsening of gait speed in both groups after the intervention, of 0.54 m/s (95% CI 0.38 to 0.70, *p* < 0.001) in the megestrol group and 0.47 m/s (95% CI 0.29 to 0.66, *p* < 0.001) in the mirtazapine group, with no difference in intergroups (0.05, 95% CI −0.15 to 0.25, *p* = 0.612). Table 5 describes the measurements of ALM, muscle strength, and physical performance.

#### 3.2.3. Adverse Events and Medication Adherence

All participants in the mirtazapine group had at least one potentially adverse effect, while the frequency of adverse effects in the megestrol group was 92.3%, with no difference in intergroups (RR 0.92, 95% CI 0.83 to 1.03, *p* = 0.145). However, the mirtazapine group had a 73% greater chance of vomiting (RR 0.27, 95% CI 0.07 to 0.99, *p* = 0.040) and a 59% greater chance of having constipation (RR 0.41, 95% CI 0.21 to 0.80, *p* = 0.009), compared to the megestrol group.

The most frequent potentially adverse effects in the mirtazapine group were drowsiness (69.2%), fatigue (65.4%), nausea (53.8%), constipation (38.5%), dyspnea (34.6%), edema (34.6%), leg weakness (30.8%), vomiting (34.6%), and dizziness (23.1%). In the megestrol group, the most frequent potentially adverse effects were fatigue (57.7%), nausea (34.6%), constipation (30.8%), edema (26.9%), dyspnea (26.9%), and leg weakness (23.1%). There was only one thromboembolic event during the study, which occurred in the mirtazapine group.

Four patients discontinued the use of the medication due to adverse effects, all in the mirtazapine group. The adverse effects reported were dyspnea, cough, and chest pain by one patient; nausea, drowsiness, and fatigue by another patient; malaise, dizziness, and nausea by two patients. In all cases, the adverse effects reported occurred in the second week of medication use, after the dose was increased.

Adherence to treatment occurred in 100% of the participants in the megestrol group and in 76.9% of the participants in the mirtazapine group. In the intergroup comparison, the megestrol group had a 31% greater chance of medication adherence compared to the mirtazapine group (RR 1.31; 95% CI 1.05 to 1.64, *p* = 0.018). For adherent participants, the mean tablet-taking was similar between the groups, being 98 ± 3% in the megestrol group and 97 ± 7% in the mirtazapine group (*p* = 0.589). At the end of the follow-up period, 54.2% of the participants in the megestrol group and 36.8% of the mirtazapine group chose to continue the treatment, with no intergroup difference (*p* = 0.598).

During the follow-up period, no reductions in chemotherapy dose were observed during the intervention, but chemotherapy was discontinued due to intolerance in three patients in the megestrol group and in five patients in the mirtazapine group, with no difference between groups (*p* = 0.702). Two patients died during the study due to disease progression, one in each group. Regarding the chance of death, there was no difference between the groups (RR 0.58, 95% CI 0.18 to 1.85, *p* = 0.361).

### 3.3. Dropouts

During the study, ten participants were lost to follow-up, eight (30.8%) in the mirtazapine group and two (7.7%) in the megestrol group. In the intergroup comparison, the mirtazapine group had an 82% greater chance of loss to follow-up compared to the megestrol group (RR 0.18; 95% CI 0.03 to 0.99, *p* = 0.040). In the mirtazapine group, four participants had medication intolerance due to adverse effects, one had a fall with a femoral fracture and required surgical osteosynthesis, two were hospitalized due to complications and progression of the oncological disease, and one died due to progression of the disease. In the megestrol group, one patient died due to disease progression and one patient was hospitalized due to severe chemotherapy toxicity.

## 4. Discussion

The main limitations of clinical research in cancer-related ACS include the use of different clinical definitions and heterogeneous subjects [10]. In the present study, only patients with diagnostic criteria for cachexia used by the international consensus were included, and the outcomes evaluated were those recommended by the literature as changes in weight, appetite, functional capacity, and body composition [1,30].

This study aimed to compare the effect of mirtazapine versus megestrol in the management of cancer-related ACS. Our main findings suggest that mirtazapine appears to be inferior to megestrol in weight and appetite improvement. In 2021, Hunter et al. [31] published that mirtazapine at a dose of 15 mg/day does not improve appetite or weight in patients with advanced cancer and depression, but the dose used was lower than that used in the present study, and the included participants had depression and a more pronounced weight loss. In our study, patients with depression were excluded.

Evidence shows that patients treated with megestrol have improved appetite and slight weight gain compared to placebo. However, weight gain tends to be modest and not clinically relevant, and it does not lead to full recovery from weight loss [23]. Uncertainties about the ideal dose and duration of treatment with megestrol also remain. Higher doses are known to be associated with greater weight gain than lower doses, however, there may be an increased risk of adverse events. In the present study, the dose of megestrol used was low and within the recommended range [10] and no thromboembolic events occurred in the megestrol group.

It is important to emphasize that the best therapeutic management of the ACS depends on the effective treatment of the neoplasm, which is not usually possible in patients with advanced oncological disease, as in the present study [32]. Despite the instituted therapeutic intervention, 50% of the participants in both groups evolved with weight loss after eight weeks of follow-up. In addition, there was a loss of functional capacity in the participants, with a significant reduction in gait speed in both groups and a significant reduction in muscle strength in men, the latter more pronounced in the mirtazapine group and not observed in women. This result demonstrates the severity of the oncological disease and the cancer-related ACS, and the importance of early identification and therapeutic intervention.

The diagnosis of ACS in cancer patients can be a challenge due to the heterogeneity of its clinical presentation [1]. In the present study, the mean BMI in both groups was compatible with normal weight at all times. Due to the obesity epidemic, the diagnosis of ACS in patients with cancer is a challenge because most patients with cancer have a normal BMI, and the classic cachexia phenotype is usually found only in patients in the refractory cachexia stage [33]. Weight loss and muscle mass loss are greater in male patients with cancer [34], and this difference may be related to the high prevalence of hypogonadism in this population [35]. In our study, we observed a higher mean weight and BMI among women.

The differences between the sexes in ACS have been little studied. Cancer-associated cachexia has been studied mainly in men, as well as in male animal models in experimental studies [36,37]. Men and women may have different susceptibility to the development and progression of cachexia or even a different response to treatment. Understanding the mechanisms that lead to such differences may allow personalized therapies as well as provide new therapeutic insights [37]. Sex hormones play important roles in maintaining skeletal muscle homeostasis [38] and there are sex differences in the inflammation of cancer-associated cachexia [39,40,41,42]. There may be a sex difference in severity and therapeutic response in ACS, and women may benefit from using mirtazapine. In addition, initial weight and BMI may interfere with treatment response. Despite the difference in therapeutic response found between the sexes, the study was not powered for this specific analysis, therefore, further studies, with an adequate sample, are needed to clarify this finding.

In the present study, there was no difference between the groups regarding the frequency of potentially adverse effects, but there was a difference regarding the medication adherence rate and loss to follow-up, with a higher adherence rate in the megestrol group and greater loss to follow-up in the mirtazapine group. All the participants who were lost to follow-up due to adverse effects were in the mirtazapine group, with a discontinuation rate due to intolerance of 15%. Overall, the most frequent side effects reported with the use of mirtazapine are drowsiness, in addition to dizziness and fatigue [43]. Among the reasons why the rate of medication adherence to mirtazapine is lower in patients with cancer compared to depressed patients, polypharmacy, and the presence of multimorbidity are highlighted, especially in older patients [44].

To the best of our knowledge, this is the first randomized, double-blind, controlled study comparing the use of mirtazapine with megestrol for the treatment of ACS in non-depressed patients with advanced cancer. Among the strengths, we highlight the fact that the study focuses on a highly prevalent clinical condition, the inclusion of non-white people, patients with anorexia, and the assessment of outcomes recommended in the literature using appropriate methodological tools. In addition, there was no difference between the groups in terms of general characteristics and regarding cancer diagnosis and treatment, and all analyses performed were adjusted for age, sex, percentage of weight loss, disease duration, staging, previous surgical treatment, and radiotherapy. It was not possible to adjust the models by type of cancer and current treatment due to the large number of categories, and consequently, the low number of participants in each of them. Among the main limitations, we mention the small sample size, the inclusion only of patients over 50 years of age, the recruitment of participants in a single center, the heterogeneity of tumors, a possible selection bias, analysis not performed by intention to treat, and the assessment of medication adherence only by indirect methods. Furthermore, the significant loss to follow-up in the mirtazapine group may have affected the power of the analysis, although the loss was more significant after eight weeks. There are some challenges associated with conducting clinical trials in patients with cancer and anorexia–cachexia syndrome due to the great vulnerability and severity of patients. Recruitment is usually low and there is difficulty in patient retention and adherence, with frequent follow-up losses that lead to delays and great difficulties in conducting clinical trials in this population [45].

## 5. Conclusions

Mirtazapine appears to be inferior to megestrol in weight and appetite improvement in patients with advanced cancer and ACS. However, there may be a difference in the therapeutic response between the sexes. Further studies are needed to clarify this finding.

## Figures and Tables

**Table 1:** General characteristics of participants (*n* = 52).

Variable	Group		*p* Value *
	Megestrol (*n* = 26) *n* (%)	Mirtazapine (*n* = 26) *n* (%)	
Sex			
Female	10 (38.5)	15 (57.7)	0.267
Male	16 (61.5)	11 (42.3)	
Race			
White	15 (57.7)	13 (50.0)	0.561
Black	0 (0.0)	2 (7.7)	
Pardo	11 (42.3)	10 (38.5)	
Asian	0 (0.0)	1 (3.8)	
Nutritional supplement	12 (46.2)	17 (65.4)	0.264
KPS performance status (%)			
60	0 (0.0)	1 (3.8)	0.281
70	5 (19.2)	6 (23.1)	
80	17 (65.4)	13 (50.0)	
90	4 (15.4)	6 (23.1)	
Primary site			
Prostate	2 (7.7)	1 (3.8)	0.400
Colon	6 (23.1)	6 (23.1)	
Upper GI tract	14 (53.8)	12 (46.1)	
Lung	2 (7.7)	7 (26.9)	
Head and neck	1 (3.8)	0 (0.0)	
Unknown	1 (3.8)	0 (0.0)	
Disease stage			
III	5 (19.2)	8 (30.8)	0.523
IV	21 (80.8)	18 (69.2)	
Current treatment			
Chemotherapy	20 (76.9)	21 (80.8)	0.668
Chemotherapy + radiotherapy	3 (11.5)	2 (7.7)	
Palliative care	0 (0.0)	1 (3.8)	
Hormone therapy	1 (3.8)	0 (0.0)	
Target therapy	0 (0.0)	1 (3.8)	
Radiological/clinical follow-up	2 (7.7)	0 (0.0)	
CHT + monoclonal antibody	0 (0.0)	1 (3.8)	

* Fisher’s exact test. Abbreviations: KPS, Karnofsky Performance Status; GI, gastrointestinal; CHT, chemotherapy.

**Table 2:** Comparative analysis between groups regarding the chance of weight and appetite improvement.

Weight						
Outcome	Raw Model			Adjusted Model *		
	RR	CI 95%	*p* Value	RR	CI 95%	*p* Value
Improvement after 4 weeks (megestrol vs. mirtazapine)	1.37	0.70; 2.65	0.36	2.69	1.09; 6.64	0.032
Improvement after 8 weeks (megestrol vs. mirtazapine)	0.84	0.41; 1.75	0.65	1.41	0.41; 4.88	0.585
**Appetite**						
**Outcome**	**Raw Model**			Adjusted Model *		
	**RR**	**CI 95%**	***p* Value**	**RR**	**CI 95%**	***p* Value**
Improvement after 4 weeks (megestrol vs. mirtazapine)	1.08	0.73; 1.59	0.70	1.21	0.82; 1.79	0.345
Improvement after 8 weeks (megestrol vs. mirtazapine)	1.65	1.07; 2.54	0.02	1.85	1.17; 2.92	0.008

* Adjusted for sex, age, disease stage, weight loss %, disease duration, previous surgical treatment, and previous radiotherapy. Abbreviations: RR, relative risk; 95% CI, 95% confidence interval.

**Table 3:** Descriptive analysis of weight and BMI in groups, by sex, before and after the intervention.

Weight (kg)				
Time	Groups	General Median (IQR)	Men Median (IQR)	Women Median (IQR)
Before the intervention	Megestrol Mirtazapine	56.7 (49.9–62.0) 56.2 (51.8–66.7)	60.6 (52.3–68.9) 52.6 (43.9–79.1)	54.8 (49.9–57.2) 57.7 (54.5–66.7)
After 4 weeks	Megestrol Mirtazapine	57.8 (51.6–60.9) 58.3 (51.5–74.5)	59.2 (51.1–65.9) 50.3 (44.1–74.5)	54.9 (51.6–57.8) 59.5 (57.8–79.5)
After 8 weeks	Megestrol Mirtazapine	57.8 (50.8–61.2) 58.8 (51.8–78.5)	59.1 (47.7–70.3) 47.5 (41.5–73.9)	57.2 (51.9–57.8) 61.2 (57.1–79.7)
**BMI (kg/m^2^)**				
**Time**	**Groups**	**General Median (IQR)**	**Men Median (IQR)**	**Women Median (IQR)**
Before the intervention	Megestrol Mirtazapine	22.9 (19.2–24.7) 23.6 (18.8–28.5)	21.7 (18.6–23.9) 18.8 (16.7–28.5)	23.5 (22.8–25.1) 24.0 (22.7–28.9)
After 4 weeks	Megestrol Mirtazapine	22.5 (19.6–24.7) 24.5 (18.4–28.7)	20.6 (19.1–23.5) 17.8 (16.7–28.2)	24.1 (23.9–24.9) 24.6 (24.0–33.1)
After 8 weeks	Megestrol Mirtazapine	22.6 (19.1–25.2) 24.8 (18.5–29.8)	21.0 (18.3–23.8) 17.5 (16.0–28.2)	25.0 (23.5–25.3) 25.0 (24.1–33.2)

Abbreviation: IQR, interquartile range.

**Table 4:** Comparative analysis between groups and times regarding the chance of scoring higher levels in the appetite score.

General						
Group × Time	Raw Model			Adjusted Model *		
	Odds Ratio	CI 95%	*p* Value	Odds Ratio	CI 95%	*p* Value
Megestrol (before vs. after 4 weeks)	16.74	6.49; 43.16	<0.01	31.45	9.64; 102.61	<0.001
Megestrol (before vs. after 8 weeks)	33.18	13.03; 84.50	<0.01	58.82	16.99; 203.58	<0.001
Mirtazapine (before vs. after 4 weeks)	7.90	3.12; 20.03	<0.01	8.92	3.65; 21.78	<0.001
Mirtazapine (before vs. after 8 weeks)	8.89	2.89; 27.35	<0.01	12.00	3.91; 36.88	<0.001
Before the intervention (megestrol vs. mirtazapine)	1.15	0.48; 2.73	0.76	1.16	0.50; 2.67	0.727
After 4 weeks (megestrol vs. mirtazapine)	0.54	0.19; 1.52	0.24	0.33	0.11; 1.02	0.054
After 8 weeks (megestrol vs. mirtazapine)	0.31	0.09; 1.06	0.06	0.24	0.07; 0.81	0.022
**Men**						
**Group × Time**	**Raw Model**			Adjusted Model *		
	**Odds Ratio**	**CI 95%**	***p* Value**	**Odds Ratio**	**CI 95%**	***p* Value**
Megestrol (before vs. after 4 weeks)	9.95	3.79; 26.13	<0.01	10.19	3.66; 28.35	<0.001
Megestrol (before vs. after 8 weeks)	34.05	10.82; 107.12	<0.01	38.61	11.38; 130.96	<0.001
Mirtazapine (before vs. after 4 weeks)	3.29	0.89; 12.19	0.07	3.17	0.95; 10.61	0.061
Mirtazapine (before vs. after 8 weeks)	2.96	0.71; 12.33	0.14	2.77	0.71; 10.84	0.143
Before the intervention (megestrol vs. mirtazapine)	1.60	0.47; 5.37	0.45	1.40	0.45; 4.35	0.558
After 4 weeks (megestrol vs. mirtazapine)	0.53	0.13; 2.18	0.38	0.44	0.10; 1.98	0.282
After 8 weeks (megestrol vs. mirtazapine)	0.14	0.03; 0.68	0.02	0.10	0.02; 0.56	0.009
**Women**						
**Group × Time**	**Raw Model**			Adjusted Model *		
	**Odds Ratio**	**CI 95%**	***p* Value**	**Odds Ratio**	**CI 95%**	***p* Value**
Megestrol (before vs. after 4 weeks)	71.72	12.17; 422.75	<0.01	97.08	13.97; 674.69	<0.001
Megestrol (before vs. after 8 weeks)	61.38	11.40; 330.45	<0.01	89.59	14.15; 567.36	<0.001
Mirtazapine (before vs. after 4 weeks)	20.60	6.20; 68.43	<0.01	25.09	7.05; 89.31	<0.001
Mirtazapine (before vs. after 8 weeks)	38.70	7.25; 206.70	<0.01	51.93	8.74; 308.39	<0.001
Before the intervention (megestrol vs. mirtazapine)	0.86	0.25; 2.92	0.81	0.96	0.27; 3.43	0.949
After 4 weeks (megestrol vs. mirtazapine)	0.25	0.05; 1.24	0.09	0.25	0.04; 1.47	0.124
After 8 weeks (megestrol vs. mirtazapine)	0.54	0.08; 3.81	0.54	0.56	0.08; 3.79	0.549

* Adjusted for age, sex, disease stage, weight loss %, disease duration, previous surgical treatment, and previous radiotherapy. Abbreviation: 95% CI, 95% confidence interval.

**Table 5:** Descriptive analysis of appendicular lean mass, hand grip strength, and gait speed in the groups.

Appendicular Lean Mass (kg)			
Time	Groups	Men Median (IQR)	Women Median (IQR)
Before the intervention	Megestrol	16.7 (15.0–20.5)	12.5 (1.5)
	Mirtazapine	15.1 (14.1–20.5)	15.6 (3.1)
After 8 weeks	Megestrol	17.4 (14.7–19.7)	12.6 (1.5)
	Mirtazapine	14.2 (13.8–20.4)	16.1 (3.6)
**Hand Grip Strength (kgF)**			
**Time**	**Groups**	**Men Median (IQR)**	**Women Median (IQR)**
Before the intervention	Megestrol	29.0 (25.0–34.0)	20.0 (18.0–22.0)
	Mirtazapine	26.0 (23.0–29.0)	23.0 (21.0–30.0)
After 8 weeks	Megestrol	27.0 (22.0–34.0)	22.0 (20.0–24.0)
	Mirtazapine	22.0 (17.0–29.0)	23.0 (22.0–26.0)
**Gait Speed (m/s)**			
**Time**	**Groups**	**Median (IQR)**	
Before the intervention	Megestrol	1.1 (0.9–1.2)	
	Mirtazapine	1.1 (0.9–1.2)	
After 8 weeks	Megestrol	0.3 (0.3–0.9)	
	Mirtazapine	0.3 (0.3–0.8)	

Abbreviation: IQR, interquartile range.

## Data Availability

All original contributions presented in this study are included in the manuscript. Further inquiries can be directed to the corresponding author.
